# Retraction: Synthesis and characterization of Mn/Co/Ti LDH and its utilization as a photocatalyst in visible light assisted degradation of aqueous Rhodamine B

**DOI:** 10.1039/c9ra90011j

**Published:** 2019-02-19

**Authors:** Andrew Shore

**Affiliations:** Royal Society of Chemistry Thomas Graham House, Science Park, Milton Road Cambridge CB4 0WF UK advances-rsc@rsc.org

## Abstract

Retraction of ‘Synthesis and characterization of Mn/Co/Ti LDH and its utilization as a photocatalyst in visible light assisted degradation of aqueous Rhodamine B’ by Priyadarshi Roy Chowdhury and Krishna G. Bhattacharyya*, RSC Adv.*, 2016, **6**, 112016–112034.

The Royal Society of Chemistry hereby wholly retracts this *RSC Advances* article following a misconduct investigation carried out by Gauhati University.

There are repeating motifs within the AFM image in Fig. 7A. Gauhati University have informed us that they have concluded that the AFM image is devoid of authenticity and has been manipulated or altered by the authors.

Signed: Andrew Shore, Executive Editor, *RSC Advances*

Date: 4^th^ February 2019

## Supplementary Material

